# Organic Hyperbolic Material Assisted Illumination Nanoscopy

**DOI:** 10.1002/advs.202102230

**Published:** 2021-08-26

**Authors:** Yeon Ui Lee, Clara Posner, Zhaoyu Nie, Junxiang Zhao, Shilong Li, Steven Edward Bopp, Gde Bimananda Mahardika Wisna, Jeongho Ha, Chengyu Song, Jin Zhang, Sui Yang, Xiang Zhang, Zhaowei Liu

**Affiliations:** ^1^ Department of Electrical and Computer Engineering University of California San Diego, 9500 Gilman Drive La Jolla CA 92093 USA; ^2^ Department of Physics Chungbuk National University Cheongju Chungbuk 28644 South Korea; ^3^ Department of Pharmacology University of California San Diego, 9500 Gilman Drive La Jolla CA 92093 USA; ^4^ Department of Mechanical Engineering University of California Berkeley CA 94720 USA; ^5^ Materials Science and Engineering University of California San Diego, 9500 Gilman Drive La Jolla CA 92093 USA; ^6^ National Center for Electron Microscopy The Molecular Foundry One Cyclotron Road Berkeley CA 94720 USA; ^7^ Materials Science and Engineering, School for Engineering of Matter Transport and Energy Arizona State University Tempe AZ 85287 USA

**Keywords:** bioimaging, organic hyperbolic materials, poly(3‐hexylthiophenes), structured illumination microscopy, super‐resolution microscopy

## Abstract

Resolution capability of the linear structured illumination microscopy (SIM) plays a key role in its applications in physics, medicine, biology, and life science. Many advanced methodologies have been developed to extend the resolution of structured illumination by using subdiffraction‐limited optical excitation patterns. However, obtaining SIM images with a resolution beyond 40 nm at visible frequency remains as an insurmountable obstacle due to the intrinsic limitation of spatial frequency bandwidth of the involved materials and the complexity of the illumination system. Here, a low‐loss natural organic hyperbolic material (OHM) that can support record high spatial‐frequency modes beyond 50*k*_0_, i.e., effective refractive index larger than 50, at visible frequencies is reported. OHM‐based speckle structured illumination microscopy demonstrates imaging resolution at 30 nm scales with enhanced fluorophore photostability, biocompatibility, easy to use and low cost. This study will open up a new route in super‐resolution microscopy by utilizing OHM films for various applications including bioimaging and sensing.

## Graphical abstract

The developed organic hyperbolic material offers an alternative path to controlling light at the nanoscale, leading to a significant resolution improvement in biological cell imaging. Organic hyperbolic material assisted illumination nanoscopy shows that precision of lateral resolution reaches down to 30 nm, which is more than 17 times spatial resolution improvement beyond the diffraction limit.

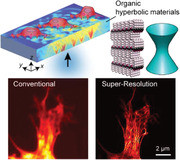

## 1 Introduction

Super‐resolution fluorescence microscopy has enabled a plethora of studies of deep subwavelength structures such as biological molecules, proteins and subcellular structures that cannot be achievable with traditional microscopes. Among various super‐resolution microscopy techniques, structured illumination microscopy (SIM) has recently attracted much attention due to its advantages in high spatial‐temporal resolution and low photo‐toxicity^[^ ^1^, ^2^ ^]^ compared to other super‐resolution techniques such as STED, PALM, and STORM.^[^ ^3^, ^4^, ^5^ ^]^ Typically, SIM can enhance the resolution about twice beyond the diffraction limit by using diffraction‐limited harmonic illumination patterns.^[^ ^6^, ^7^ ^]^ Yet one requirement of standard SIM is the precise knowledge of the illumination patterns to reconstruct a super‐resolution image and distortion in illumination will usually cause reconstruction artifacts.^[^ ^8^ ^]^ To alleviate such a limitation, speckle‐structured illumination microscopy (speckle‐SIM) has recently been developed^[^ ^8^, ^9^ ^]^ with the consideration of a statistical prior that the averaged speckle illuminations are homogeneous.^[^ ^8^ ^]^ However, resolution improvement of speckle‐SIM is still about twofold as the speckle patterns are typically also diffraction limited.

**Figure 1:**
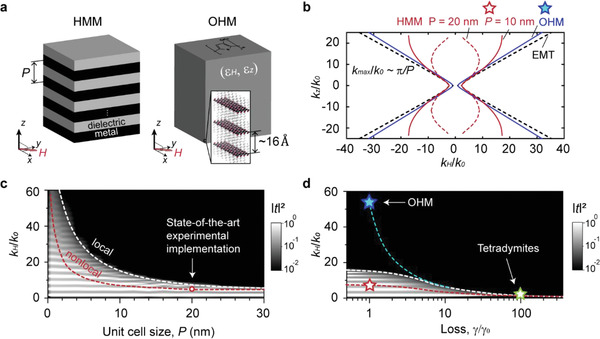
Comparison of various hyperbolic materials in the visible spectral range. a) Schematic figures of a planar HMM (left panel) and a natural organic hyperbolic material (OHM) (right panel). b) Calculated optical dispersion of the multilayer HMM (Ag/SiO_2_, 50% metal filling ratio) for unit cell size *P* = 20 nm (red dashed), *P* = 10 nm (red solid), effective medium theory (EMT) (black dashed) for *λ* = 470 nm; optical dispersion of OHM used in this work (blue) for *λ* = 470 nm. c) Transmission (|*t*|^2^) as a function of normalized wavevector *k*_H_/*k*_0_ and unit cell size *P* in the HMM. The total slab thickness is maintained the same (182 nm) in each case, while the number of individual layers is adjusted. White dashed curve for the local description (Equation S1, Supporting Information) and red dashed curve for the nonlocal description (Equation S4 , Supporting Information) correspond to cutoff wavevectors, *k*_max_/*k*_0_ when |*t*|^2^_(k‐cutoff)_ = 0.01. d) Transmission (|*t*|^2^) as a function of *k*_H_/*k*_0_ and loss (*ε*_H_′′), *γ*/*γ*_0_, where *γ*_0_ = 0.03. For comparison, the cutoff wavevector of the HMM with *P* = 10 nm (red star, highly challenging to make a smooth and low‐loss HMM^[^^]^), tetradymites (green star, adapted from the ref. ^[^ ^39^ ^]^), and OHM (cyan star, in this study) are also shown.

To further improve the resolution capability of SIM, a material system that supports higher spatial bandwidth needs to be sought. For example, plasmonic‐structured illumination microscopy (plasmonic‐SIM)^[^ ^10^, ^11^, ^12^ ^]^ utilizing plasmonic waves as illumination sources has further extended the resolution beyond the conventional SIM. Metamaterial is another platform people used to achieve super resolution.^[^ ^13^, ^14^, ^15^ ^]^ One of the most widely used metamaterials is metal and dielectric multilayer hyperbolic metamaterial (HMM)^[^ ^16^, ^17^, ^18^ ^]^ (**Figure** **1**a, left), implemented as the first hyperlens by curving the multilayers and converting the evanescent waves from an object directly into propagating waves.^[^ ^19^, ^20^, ^21^, ^22^, ^23^ ^]^ In another aspect, HMMs can be also implemented to provide illumination patterns far beyond the diffraction limit.^[^ ^14^, ^24^, ^25^ ^]^ Therefore, with such high‐*k* illuminations, the metamaterial assisted illumination nanoscopy (MAIN) currently represents a very unique SIM approach with greatly extended resolving power.^[^ ^14^, ^26^ ^]^ Challenge still remains to obtain images with a resolution beyond 40 nm at visible frequency.

In this Letter, we demonstrate a new OHM‐based SIM method with 30 nm resolution capability. With periodicity at 1.6 nm, hyperbolic dispersion of the developed OHM film supports remarkably high‐*k* optical modes in the visible frequency. Comparing to inorganic counterparts, the OHMs not only provide better deep subwavelength‐scale illuminating field confinement in visible spectral range, but also offer many advantages such as extremely enhanced photostability of fluorophores near the OHM,^[^ ^27^ ^]^ easy fabrication process, and excellent biocompatibility^[^ ^28^, ^29^ ^]^ for bioimaging applications.

Figure 1b shows the calculated optical dispersion of an Ag‐based HMM using the effective medium theory (EMT) where its isofrequency surface, with the open hyperbola topology,^[^ ^30^, ^31^ ^]^ can be clearly seen in *k*‐space. Note that the maximum available wavevector and photonic local density of states (LDOS)^[^ ^32^, ^33^ ^]^ are limited because the EMT description of the HMM eventually fails when taking into account the limited unit cell size as shown in Figure 1b,c (see Supporting Information S1 for details). Besides, optical losses and nonlocal response^[^ ^34^, ^35^ ^]^ limit the maximum available wavevector of propagating optical modes as shown in Figure 1c,d. Natural hyperbolic materials^[^ ^36^, ^37^, ^38^ ^]^ could circumvent some of the limitations on the HMMs, e.g., the finite structural periodicity and nonlocal effect. For example, Bi_2_Se_3_, Bi_2_Te_3_,^[^ ^39^ ^]^ and GaTe^[^ ^40^ ^]^ have been experimentally demonstrated as the natural hyperbolic materials. Unfortunately, these materials are very lossy leading to hyperbolic modes with a very small wavevector (Figure 1d, green star)—much worse than that provided by the Ag‐based HMMs (Figure 1d, red star). Compared to the traditional HMMs and other natural hyperbolic materials, the OHMs used in this work (Figure 1a, right) feature a low optical loss^[^ ^41^ ^]^ and thus support record high‐*k* modes (Figure 1d, cyan star). Therefore, the OHM presents an excellent material platform achieving the highest possible resolution improvement for super‐resolution microscopy.

## 2 Results and Discussion

**Figure 2:**
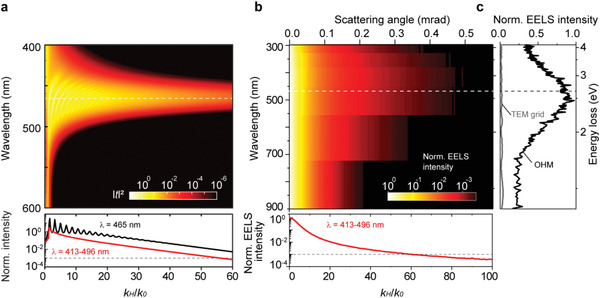
Optical properties of an OHM made by P3HT. a) Calculated OTF (i.e., normalized transmitted intensity (|*t*|^2^) as a function of *k*_H_/*k*_0_) of a 180 nm thick OHM film. Bottom inset: OTF of the OHM film at *λ* = 465 nm (black curve) and averaged OTF in the wavelength range of 413–496 nm (red curve). b) Color map of *k*‐EELS spectra over a range of different scattering angles (momentum) are shown. Bottom inset: normalized EELS intensity as a function of *k*_H_/*k*_0_ in the wavelength range of 413–496 nm. c) *k*‐EELS spectra integrated over the scattering angles for a bare TEM grid (gray curve) and the OHM film (black curve), respectively.

To produce the OHM, 100 mg of 98% regioregular poly(3‐hexylthiophene‐2,5‐diyl) (rr‐P3HT) was dissolved in 1 mL chlorobenzene (CB) solution (P3HT:CB = 100 mg mL^−1^). From this solution, thin films were spin‐coated on top of a 0.17 mm thick plasma‐cleaned glass coverslip as described in reference^[^ ^41^ ^]^ (see the Experimental Section for details of the film fabrication). The fabricated 182 nm thick rr‐P3HT films exhibit a hyperbolic dispersion in the visible spectral range 400–560 nm.^[^ ^41^ ^]^ The capability of super‐resolution imaging using the OHM film is estimated by the measured permittivity (see details in Supporting Information S2, Figure S2) and resultant optical transfer function (OTF) based on transmission coefficient calculation (see details in Supporting Information S1, Equation S5)^[^ ^32^, ^42^ ^]^ (**Figure** **2**a). The OTF of the OHM has an exceptionally high cutoff spatial‐frequency *k*_cutoff_ = 54*k*_0_ when OTF_(_ *_k_*_‐cutoff)_ = 0.01 at *λ* = 465 nm. The wavelength of the propagating waves and thus the resolution supported by this OHM can reach below 10 nm. A comprehensive comparison of HMM multilayers in various combinations with the same cutoff spatial‐frequency (see a detailed comparison in Supporting Information S3) reveals that the metal film thickness has to be around 1 nm scales introducing significant fabrication challenges. In addition, the optical property of such thin metallic film has to be modified by nonlocal or quantum size effect, which makes the achievement of such OTF highly questionable. Momentum‐resolved electron energy loss spectroscopy (*k*‐EELS)^[^ ^43^ ^]^ measurement in a transmission electron microscope (TEM) further confirms the high‐*k* mode excitation in the OHM. Figure 2b shows the measured energy‐momentum (scattering angle) dispersion of the OHM at 200 keV incident energy of electron (see the Experimental Section for details of the *k*‐EELS measurement). The *k*‐EELS spectra integrated over the scattering angles (Figure 2c and Figure S4, Supporting Information) show additional evidence of excitation of the high‐*k* hyperbolic polariton modes at the hyperbolic dispersion wavelength range 400–560 nm (2.2–3.1 eV).

The high‐*k* illumination brings super‐resolution to a traditional microscopy.^[^ ^44^ ^]^ We tested the imaging performance of the OHM assisted illumination nanoscopy with 20 nm diameter fluorescent beads as imaging objects. The fluorescent beads (see details in the Experimental Section) were drop‐casted on the OHM‐coated coverslip substrate. It has been shown previously that the high‐*k* speckle patterns on the top surface of hyperbolic materials can be generated and controlled by tuning the incident non‐uniform illumination patterns.^[^ ^14^, ^24^ ^]^ In the experimental setup (see details in the Experimental Section and Supporting Information S5), the high‐*k* speckle illuminations on the OHM were varied by adding mechanical forces on the multi‐mode fiber with a stepping motor. At the sample plane, the high‐contrast and high‐*k* speckles excited the fluorophores in a specimen, and after passing through an emission filter (520/40 nm band‐pass), the fluorescence signal was collected by an sCMOS camera. By varying the speckle illumination *N*‐times, *N*‐different fluorescence image frames were obtained via an 80×/0.6 NA objective lens. A set of *N* = 300 frames (5 frames per second) were captured in the whole sequence, which were used to reconstruct the final super resolution image by the Blind‐SIM reconstruction algorithm^[^ ^8^ ^]^ (see the Experimental Section for details of the reconstruction).

**Figure 3:**
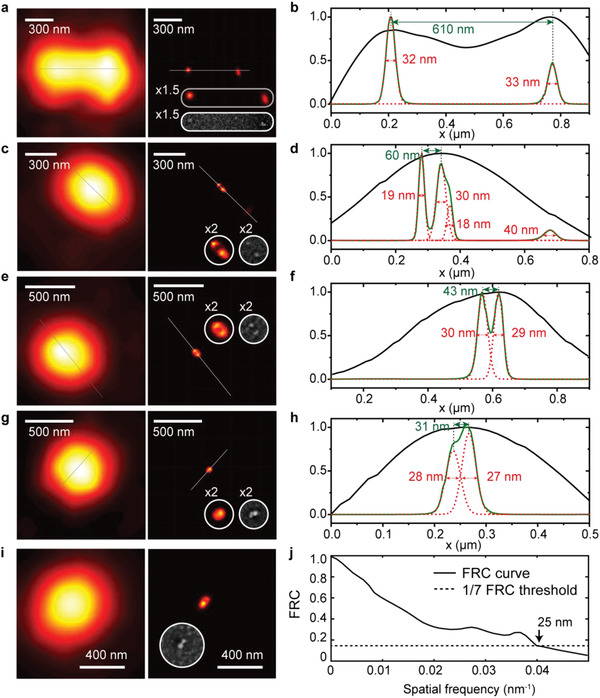
Images of fluorescent beads with the averaged diameter of 20 nm. The diffraction‐limited images (left panel), the super‐resolution images and scanning electron microscope (SEM) images (middle panel) of fluorescent beads, and the cross‐section (white solid line) curves (right panel) are plotted for two adjacent beads with the center‐to‐center distance of a,b) 610 nm, c,d) 60 nm, e,f) 43 nm, and g,h) 31 nm, respectively. i) Diffraction limited image and reconstructed image of two fluorescence beads (center‐to‐center distance of 23 nm). j) The standard 1/7 FRC resolution criteria illustrates 25 nm Fourier space cutoff. Objective lens: 80×/0.6 NA. Exposure time: 500 ms. Total frame: 500.

**Figure** **3** shows the diffraction‐limited image (left panel) and super‐resolution image (middle panel) of fluorescence beads. As can be seen, the super‐resolution image reveals much greater details that cannot be observed by any individual diffraction‐limited image. The cross‐section (white solid line) curves are plotted (right panel) for two adjacent beads with the center‐to‐center distance of 610 nm (Figure 3a,b), 60 nm (Figure 3c,d), 43 nm (Figure 3e,f), and 31 nm (Figure 3g,h), respectively. After fluorescence imaging, the imaged locations were fixed for SEM by using a reference marker on the sample. The distributions of fluorescent beads are identified by SEM images (insets of the middle panel of Figure 3). To confirm the actual resolution achieved, a Fourier ring correlation (FRC)^[^ ^45^ ^]^ analysis was subsequently performed. The FRC is a resolution criterion that is widely accepted for super‐resolution microscopy. Two reconstructed images based on two independent subsets of data were used. The standard 1/7 FRC resolution criterion illustrates that 25 nm resolution has been achieved (see Figure 3j), representing more than 17 times spatial resolution improvement beyond the diffraction limit.

**Figure 4:**
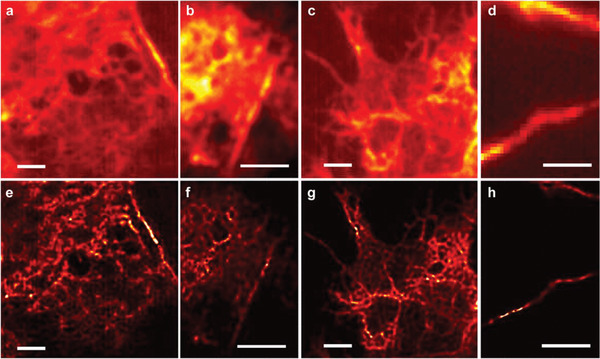
Venus‐CAAX tagged Cos‐7 cell membrane images. a–d) Conventional wide‐field image. e–h) Super‐resolution images. Scale bar: 2 µm.

To test the biocompatibility, we present reconstruction results of fluorescently labeled plasma membrane and actin filaments in Cos‐7 cells. Cells were grown on top of the OHM substrate and then transiently transfected with fluorescently labeled F‐actin (Lifeact‐Venus) or a fluorescent protein targeted to the plasma membrane using the C‐terminal CAAX sequence of KRas (Venus‐CAAX) (see the Experimental Section for Cos‐7 cell transfection). In the imaging experiments, 200 frames were acquired with 10 W cm^−2^ illumination intensity. **Figure** **4**a–d shows the diffraction‐limited images of Venus targeted to the plasma membrane in Cos‐7 cells. The corresponding reconstructed images are shown in Figure 4e–h.

**Figure 5:**
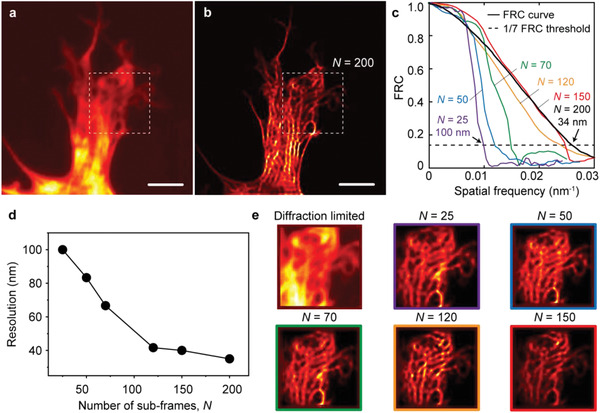
Venus‐lifeact tagged Cos‐7 cell actin images. a) Conventional wide‐field images. b) Super‐resolution images. Scale bar: 2 µm. c) FRC resolution criteria curves generated from two reconstructed images based on two independent subsets of data with the number of subframes, *N*. The standard 1/7 FRC resolution criteria illustrates 34 nm Fourier space cutoff for *N* = 200. d) The standard 1/7 FRC resolution criteria illustrate the *N*‐dependent resolution of the reconstructed images. e) Reconstructed images generated from *N* subframes.

**Figure** **5** shows diffraction‐limited images (Figure 5a) and super‐resolution images (Figure 5b) of Venus labeled actin in Cos‐7 cells via our super‐resolution imaging technique. The FRC analysis and resolution of the reconstructed images (outlined by a white box in Figure 5b) as a function of the number of subframes, *N*, used to generate a super‐resolution image are shown in Figure 5c–e. The resolution of ≈40 nm scales is achieved by only 120 frames (Figure 5d) for a 5 fps acquisition rate. The nature of a stochastic blinking of fluorophores is one of the main factors in limiting the temporal resolution of probe‐based localization microscopy. On the other hand, predetermined speckles in OHM assisted illumination microscopy can represent much more prior information enabling improved image reconstruction with less measurements. This eventually can lead to higher imaging speed. Note that during the image acquisitions, almost negligible photobleaching was observed because of the significantly reduced photobleaching rates by hyperbolic optical modes supported by the OHM due to the large resultant Purcell effect.^[^ ^27^ ^]^

After the optical imaging, the samples were dried and random locations were selected to confirm that the OHM is a chemically stable and biocompatible substrate for cell transfection. The OHM is a conductive substrate to uniquely enable scanning electron microscopy (SEM) imaging of Cos‐7 cell without sealing additional layers on top of the cells. An SEM image of Cos‐7 cells cultured on a glass coverslip (Figure S6a, Supporting Information) shows overly bright and unstable intensity due to accumulated charges on the top surface of the glass. In contrast, SEM images of Cos‐7 cells cultured on the electrically conductive and biocompatible OHM substrate (Figure S6b–f, Supporting Information) show outstanding resolution and contrast over the entire field of view.

## 3 Conclusion

In summary, we demonstrated an OHM‐based super‐resolution imaging technique based on the speckle structured illumination microscopy. The achieved precision of lateral resolution reaches down to 30 nm, which is more than 17 times spatial resolution improvement beyond the diffraction limit. The developed OHM offers an alternative path to controlling light at the nanoscale, leading to a significant resolution improvement in biological cell imaging. We observed that our OHM substrate is highly stable for cellular imaging after cell transfection and no protection layer was required for cell culture, well surpassing the capability of traditional hyperbolic materials. Our OHM‐based imaging technique will open up a new paradigm to utilize the OHM films instead of glass coverslips for microscope slides to achieve enhanced fluorophore stability, biosample friendly, easy to fabricate, and low cost for high‐resolution imaging applications.

## 4 Experimental Section

### OHM Film Fabrication

98% regioregular P3HT (molecular weight average *M*_w_ ≈ 87 000, Sigma‐Aldrich) 100 mg were dissolved in 1 mL chlorobenzene by heating the solution to 50 °C for 3 h and resting at room temperature for 2 h, resulting in a dark orange‐colored solution. Thin films were spin‐coated on 0.17 mm thick clean glass coverslips at 5000 rpm for 60 s. The film thickness of 182 nm was measured by spectroscopic ellipsometry. Please see Supporting Information S2.

### Electron Energy Loss Spectroscopy (EELS) Measurement

Thin films of rr‐P3HT (10 and 100 mg mL^−1^ concentrations) were prepared on glass substrates. Argon reactive‐ion etching was performed on an Oxford Instruments Plasmalab 80 Plus to reduce the thickness of the films. The etching condition was set to be 40 sccm Argon with 100W RF power. Film thicknesses of 28 and 26 nm were measured with complex permittivity by VASE. These thin films were collected onto carbon‐coated TEM grids after they were floated off by the dissolution of the substrates in water. High EELS measurement was carried out using transmission electron microscopy (TEM) on the FEI monochromated F20 UT Tecnai, operating at 200kV, located in the National Center for Electron Microscopy (NCEM) at Molecular Foundry, Lawrence Berkeley National Laboratory (LBNL). This microscope was equipped with Gatan Imaging Filter (GIF) along with a monochromator incorporated into the gun, therefore, it permitted electron energy loss spectroscopy to be performed with an energy resolution of ≈0.2 eV. This high spectral resolution enabled the characterization of the rr‐P3HT film in the electron energy‐loss range close to the zero‐loss peak with minimum overlapping. Please see Supporting Information S3. Electrons with kinetic energies *E*_o_ and momenta ℏ*k*_o_ passed through thin films, where the electrons would excite hyperbolic optical mode inside the film. They were scattered inelastically into angles *θ* losing energies ∆*E*≪*E*_o_ and transferring momenta ℏ*k* to the film. The momentum transferred to the surface in the case of normal incidence of the incoming electrons amounted to *k*_o_ *θ*. An 1 eV energy slit was inserted on GIF to measure the scattering angle of a small portion of energy filtered electrons. The energy filter was moved along the loss spectrum with a 0.5 eV step size to observe scattering angle as a function of selected energy loss portion. Note that the scattering angles larger than 0.35 mrad correspond to momentum larger than 60*k*_0_.^[^ ^46^ ^]^

### Fluorescent Beads for Imaging

The fluorescent beads (Thermo Fisher Scientific, FluoSpheres Sulfate Microspheres, yellow‐green fluorescent (505/515), 2% solids) were drop‐casted on the OHM‐coated coverslip substrate. After acquiring the fluorescence images, the SEM images of the same regions were acquired.

### Cos‐7 Cell Transfection

Cos7 cells were cultured in Dulbecco modified Eagle medium (DMEM; Gibco) containing 4.5 g L^−1^ glucose and supplemented with 10% (v/v) fetal bovine serum (FBS, Sigma) and 1% (v/v) penicillin‐streptomycin (Pen‐Strep, Sigma‐Aldrich). All cells were maintained in a humidified incubator at 37 °C with a 5% CO2 atmosphere. 24 h prior to transfection, cells were seeded onto OHM substrate and grown to 50–70% confluence. Cells were then transfected with 100 ng of pcDNA3‐Lifeact‐Venus (87613, Addgene) for actin‐labeling or 100 ng of pcDNA3‐Venus‐CAAX (87612, Addgene) for plasma membrane labeling using Lipofectamine 2000 (Invitrogen) and grown an additional 24 h before fixation. Cells were washed with Phosphate‐buffered saline (PBS) before fixation with 4% paraformaldehyde and 0.2% glutaraldehyde PBS for 10 min at room temperature. Cells were quickly rinsed in PBS after fixation and quenched with freshly made 0.1% NaBH4 ice‐cold PBS. After quenching, cells were washed three times for five min each with PBS on a shaker. Cells were imaged at room temperature and stored in PBS at 4 °C.

### Experiment Setup

A 488 nm laser (Coherent Genesis) was coupled into a multimode fiber (Thorlabs, core diameter: 50 µm, NA 0.2). The other end of the fiber was coupled to a reflective fiber collimator that was attached to a custom‐made adapter of the microscope condenser. The fiber end was imaged to the OHM film, projecting diffraction‐limited speckle patterns. The OHM film converted the pattern into high‐resolution speckles that illuminated the object on the other side. The speckle was controlled by a step motor which will stretch the fiber spool during image acquisition. An sCMOS camera was used for imaging acquisition (Hamamatsu ORCA Flash 4.0 v3). To synchronize all equipment properly, Matlab software was used to control a DAQ voltage output module (NI‐9263) from National Instruments. Please see Supporting Information S4.

### Imaging Reconstruction

All of the image processing and reconstruction were performed in MATLAB. An iterative reconstruction algorithm, blind‐SIM^[^ ^8^ ^]^ which does not require exact knowledge of the illumination pattern, was used to retrieve the object information. For blind‐SIM, an assumption was made that all illumination patterns add up to a uniform pattern. Both the object and the illumination patterns were treated as unknowns in real space and were solved using a cost‐minimization strategy. Each super‐resolution frame was reconstructed from multiple subframes. The GPU‐based reconstruction typically takes 10–30 min on a Nvidia GTX 1080 Ti.^[^ ^14^ ^]^

## Conflict of Interest

The authors declare no conflict of interest.

## Supporting information

Supporting Information

## Data Availability

The data that support the findings of this study are available from the corresponding author upon reasonable request.
