# Supplementary material for: Organic Hyperbolic Material Assisted Illumination Nanoscopy
Source: Adv Sci (Weinh). 2021 Aug 26;8(22):2102230. doi: 10.1002/advs.202102230 (PMC8596137; doi:10.1002/advs.202102230)
Supplement: Supplementary file 1 — Supporting Information [file ADVS-8-2102230-s001.pdf]

© 2021 Wiley-VCH GmbH

ADVANCED SCIENCE
Open Access

Supporting Information

for *Adv. Sci.*, DOI: 10.1002/advs.202102230

Organic hyperbolic material assisted illumination nanoscopy

*Yeon Ui Lee, Clara Posner, Zhaoyu Nie, Junxiang Zhao, Shilong Li, Steven Edward Bopp, G. Bimananda M. Wisna, Jeongho Ha, Chengyu Song, Jin Zhang, Sui Yang, Xiang Zhang, Zhaowei Liu**

Supporting Information

Organic hyperbolic material assisted illumination nanoscopy

*Yeon Ui Lee, Clara Posner, Zhaoyu Nie, Junxiang Zhao, Shilong Li, Steven Edward Bopp, G. Bimananda M. Wisna, Jeongho Ha, Chengyu Song, Jin Zhang, Sui Yang, Xiang Zhang, Zhaowei Liu**

Contents:

S1. Local and nonlocal optical responses of HMM

S2. Characterization of OHM

S3. HMM multilayers in various combinations with cut-off spatial-frequency of 54k_0

S4. EELS measurement

S5. OHM assisted high-*k* illumination microscopy

S6. SEM images of Cos-7 cells

S1. Local and nonlocal optical responses of HMM

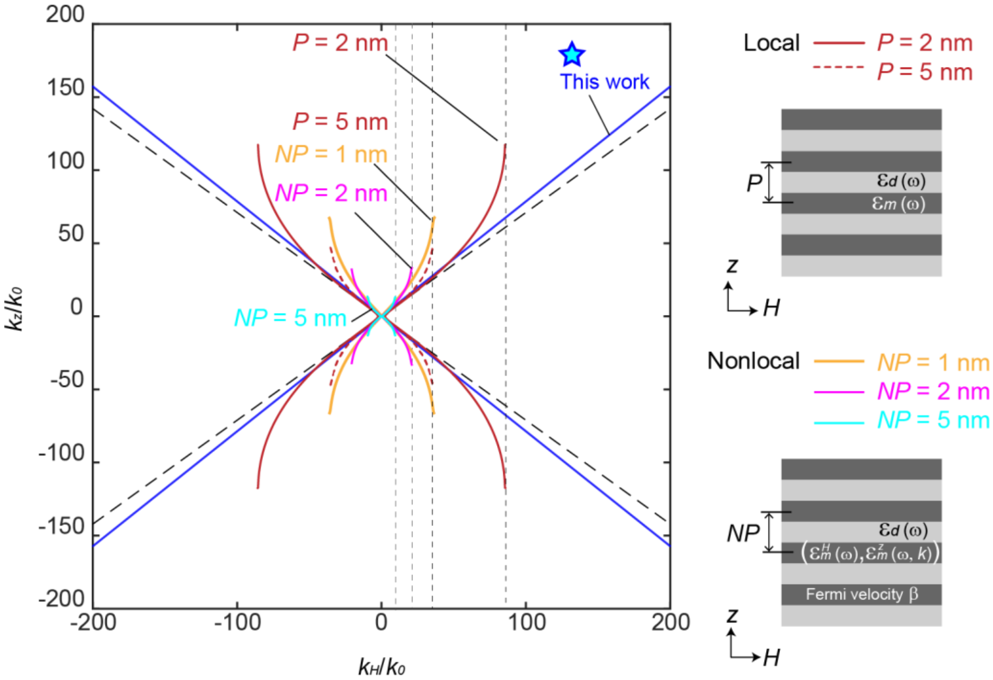

Fig. S1 | Local and nonlocal models for hyperbolic dispersion. Dispersion curves of the HMM for λ = 470 nm. The filling factor of the HMM is 0.5. Material parameters for Ag: $\hbar\omega_p$=8.8 eV, $\hbar\gamma$ = 0.07 eV, and v_F = 1.39× 10^6 m/s.

(1) Local model[1]

In Fig. S1, we consider an infinite periodic Ag/SiO_2 HMM where Ag layers having permittivity ε_m and thickness d_m, and SiO_2 layers with permittivity ε_d and thickness d_d (periodicity $P = d_m + d_d$). For the dispersion relation, we label the wavevectors by a real tangential component k_H lying in the *x*–*y* plane, and Bloch wavevector k_z. The relation between k_H and k_z is given by Bloch's theorem:

$$\cos[k_z P] = \cos(w_m d_m)\cos(w_d d_d) - \left[\frac{(\varepsilon_d w_m)^2 + (\varepsilon_m w_d)^2}{2(\varepsilon_d w_m)(\varepsilon_m w_d)}\right]\sin(w_m d_m)\sin(w_d d_d) \qquad \text{(S1)}$$

where $w_i = \sqrt{\varepsilon_i \omega^2/c^2 - k_H^2}$; $i = m, d$ is the z-component of the wave vector in the metal or dielectric. Expanding Eq. (S1) up to $(w_i d_i)^2$ reduces it to the hyperbolic dispersion of effective medium theory (EMT):

$$\omega^2/c^2 = k_z^2/\varepsilon_H + k_H^2/\varepsilon_z \tag{S2}$$

where $\varepsilon_H = (\varepsilon_m d_m + \varepsilon_d d_d)/(d_m + d_d)$, and $\varepsilon_z = \varepsilon_m \varepsilon_d (d_d + d_m)/(\varepsilon_m d_d + \varepsilon_d d_m)$. The exact results of Eq. (S1) are shown in Fig. S1 for $P = 2$ nm and $P = 5$ nm. The exact dispersions show deviations from EMT (black dashed curve) as k_H increases. The cut-off in the range of possible k_H, $k_{H\,cut-off} \propto 1/(d_d + d_m)$ puts a limit on the LDOS.

(2) Nonlocal model[2]

The nonlocal model has been proposed to include the effect of electron-electron repulsion in metals when metal feature sizes become comparable to the Fermi screening length. This repulsion spatially smears out the charge distribution, and charge penetration leads a spatially dispersive dielectric function. From the hydrodynamic Drude theory, the free-electron nonlocal optical response of metals gives rise to a large-wavevector cutoff in the dispersion that is inversely proportional to the Fermi velocity of the electron gas. In the hydrodynamic Drude model, the metal supports both transverse and longitudinal waves. In terms of the permittivity:

$$\varepsilon_m^T(\omega) = 1 - \omega_p^2/(\omega^2 + i\omega\gamma) \tag{S3a}$$

$$\varepsilon_m^L(k,\omega) = 1 - \omega_p^2/(\omega^2 + i\omega\gamma - \beta^2 k^2) \tag{S3b}$$

Here, γ is the Drude damping, ω_p is the plasma frequency, and the nonlocal parameter $\beta = \sqrt{3/5}v_F$ where v_F is the Fermi velocity. The additional longitudinal waves fundamentally change the system's optical response.

For an arbitrary unit cell (that is metamaterial unit repeat or identity distance) size P, the exact dispersion relation for the infinite HMM is given by:

$$\cos\theta_b = \left\{\cos\theta_b\left[k_L\cos\theta_m\sin\theta_l - k_H\frac{(w_d-w_m)}{z_m}\sin\theta_m\cos\theta_l\right] + \sin\theta_d\left[k_H\frac{(w_d-w_m)}{z_d}(1-\cos\theta_m\cos\theta_l) - \frac{1}{2}\left[\frac{k_H^2}{k_L}\frac{(w_d-w_m)^2}{z_d z_m} + k_L\left(\frac{z_d}{z_m}+\frac{z_m}{z_d}\right)\right]\sin\theta_m\sin\theta_l\right]\right\}\left[k_L\sin\theta_l - k_H\frac{(w_d-w_m)}{z_m}\sin\theta_m\right]^{-1}$$

(S4)

with $\theta_b = k_z P$, $\theta_d = k_{dz}d_d$, $\theta_m = k_{mz}^T d_m$, $\theta_l = k_{mz}^L d_m$, $z_d = k_{dz}/(k_0\varepsilon_d)$, $w_d = k_H/k_0$, $z_m = k_{mz}^T/(k_0\varepsilon_m^T)$, $w_m = k_H/(k_0\varepsilon_m^T)$, where $(k_{mz}^L)^2 + k_H^2 = k_L^2$ with $k_L^2 = (\omega^2 + i\omega\gamma - \omega_p^2)/\beta^2$.

(3) Transmission through planar hyperbolic media[3]

We assume that the hyperbolic material slab is embedded in a uniform medium of constant permittivity (ϵ). In such a medium, the dispersion relation is $k_H^2 + k'^2_z = k_0^2\epsilon$. The transmission coefficient for TM waves is

$$t(k_H,\omega) = \frac{2}{2\cos k_z d - i\left(\frac{k'_z\epsilon_H}{k_z\epsilon} + \frac{k_z\epsilon}{k'_z\epsilon_H}\right)\sin k_z d} \qquad \text{(S5)}$$

, where the dispersion relations ((S1), S(2), S(4), or even something else) can be used to define k_z in terms of k_H and ω.

S2. Characterization of OHM

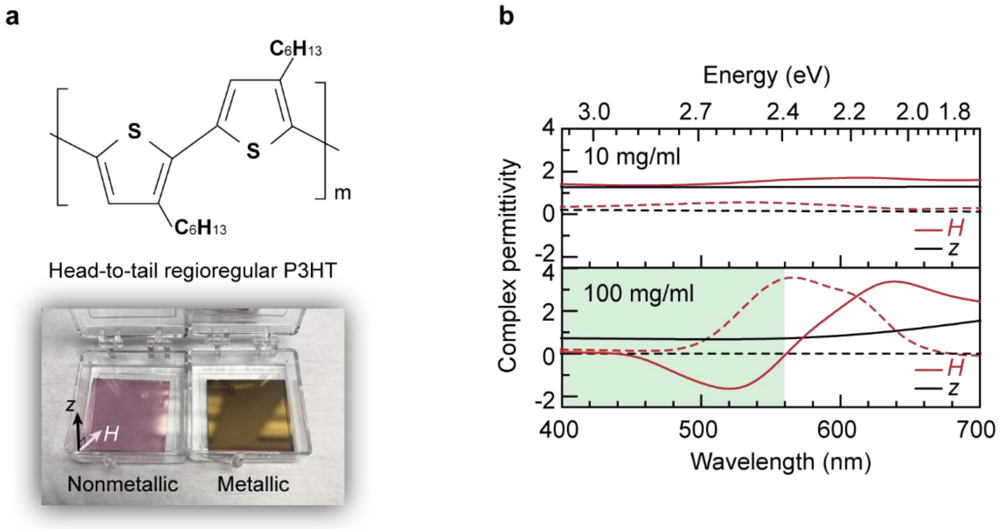

Fig. S2 | Optical properties of OHM. (a) photograph of rr-P3HT films fabricated with different concentration rr-P3HT in chlorobenzene solution. (b) The spectral dependence of the real (ϵ_{1x} (black solid curve) and ϵ_{1z} (red solid curve)) and imaginary (ϵ_{2x} (black dashed curve) and ϵ_{2z} (red dashed curve)) parts of the permittivity for different concentration of rr-P3HT film. (Thickness: 28 and 182 nm for 10 and 100 mg/ml concentration films, respectively.)

Ellipsometry measurement. A rotating polarizer type spectroscopic ellipsometer (J.A. Woollam M-2000D, J. A. Wollam Co. Ltd.) was used to obtain the permittivity of OHM as a function of photon energy. The measurements were performed in the spectral range of 1.55–4.0 eV (310-800 nm) for angles of incidence 60° and 70°. This approach improves the accuracy of modeling analysis, allowing determination of film thicknesses and refractive index values. The measured ellipsometric constants Ψ and Δ are defined from the ratio of the reflection coefficients r_p and r_s for the *p*- and *s*- polarization, respectively. Beam size: 3 mm.

S3. HMM multilayers in various combinations with cut-off spatial-frequency of 54k_0

For the total thickness of 182 nm, OHM supports a cut-off spatial-frequency of 54k_0 when $OTF_{(k\text{-cut-off})}$ = 0.01 at λ = 465 nm. Exemplary HMM multilayers supporting the same cut-off spatial-frequency of 54k_0 are shown below, based on dispersion curve calculation with the local model (Supporting Information S1). Nevertheless, fabrication of such HMMs is a big challenge.

HMM multilayers, materials	Metal filling ratio	Period (nm)	Number of periods
Ag/SiO_2	30 %	2.7	68
	50 %	1.9	96
	70 %	1.0	182
Ag/Al_2O_3	30 %	2.2	82
	50 %	2.7	68
	70 %	- (no existence)	-
Ag/TiO_2	30 %	- (no existence)	-
	50 %	3.0	60
	70 %	- (no existence)	-
Au/Al_2O_3	10-90 %	- (no existence)	-
Au/TiO_2	10-90 %	- (no existence)	-

Fig. S3 | HMM multilayers in various combinations with cut-off spatial-frequency of 54k_0

S4. EELS measurement

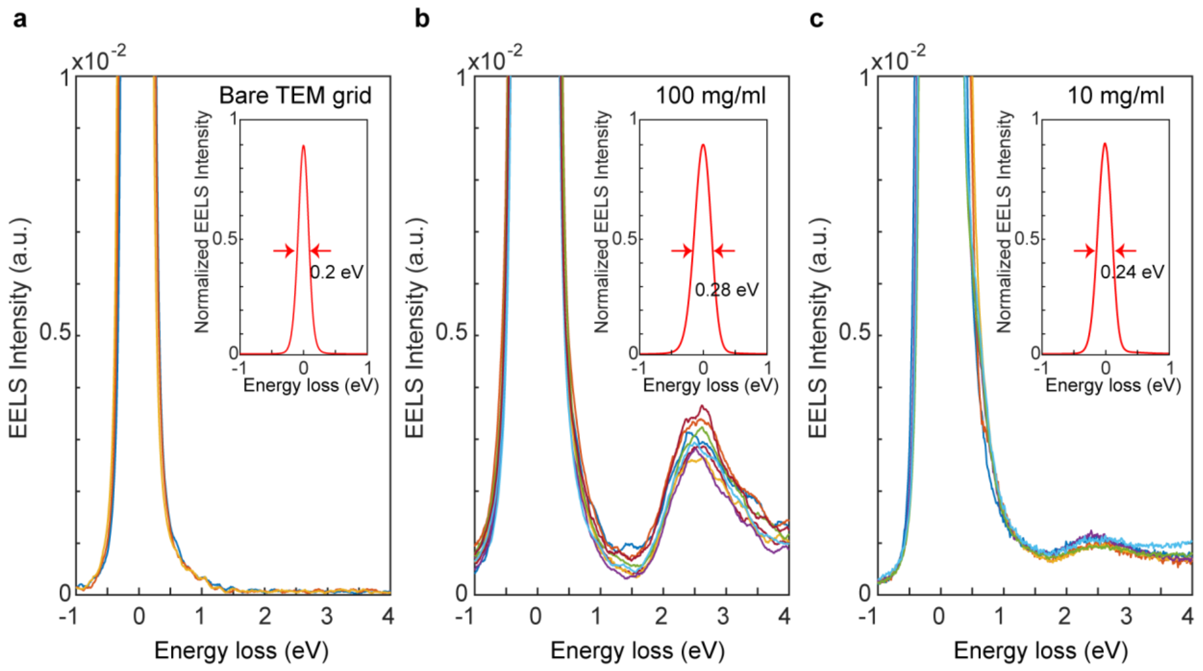

Fig. S4 | EELS spectra measured at several different sample positions of (a) bare TEM grid, (b) P3HT films having a high-concentration; OHM (100 mg/ml) and (c) a low-concentration; dielectric (10 mg/ml). The films thicknesses of 26 nm and 28 nm were measured by VASE.

In EELS, the zero loss peak (ZLP) is associated with electrons that have lost no energy as they have passed through the sample, and the full-width at half maximum of ZLP is used as a measure of the spectral resolution. The measured energy resolutions were in the range of 0.2 – 0.3 eV (see insets in Fig. **S4 a-c**.). A greater energy loss in the OHM film (**Fig. S4b**) is evident, indicating the excitation of high-*k* hyperbolic modes in the range 2.2 – 3.1 eV (400 – 560 nm). The high degree of crystallinity and the resulting strong transition's oscillator strength of rr-P3HT have a tremendous effect on hyperbolic dispersion (**Fig. S2b**) and high-*k* mode excitations. To confirm the crystallinity-dependent high-*k* mode excitations, we also performed EELS measurement for the low concentration rr-P3HT film (10 mg/ml), which has lower crystallinity (**Fig. S4c**). The reduction of the EELS intensity in the range of 2.2 – 3.1 eV

indicates relatively weak transition's oscillator strength in optical dispersion, which leads to an absence of a negative real part of permittivity as shown in **Fig. S2b**.

S5. OHM assisted high-*k* illumination microscopy

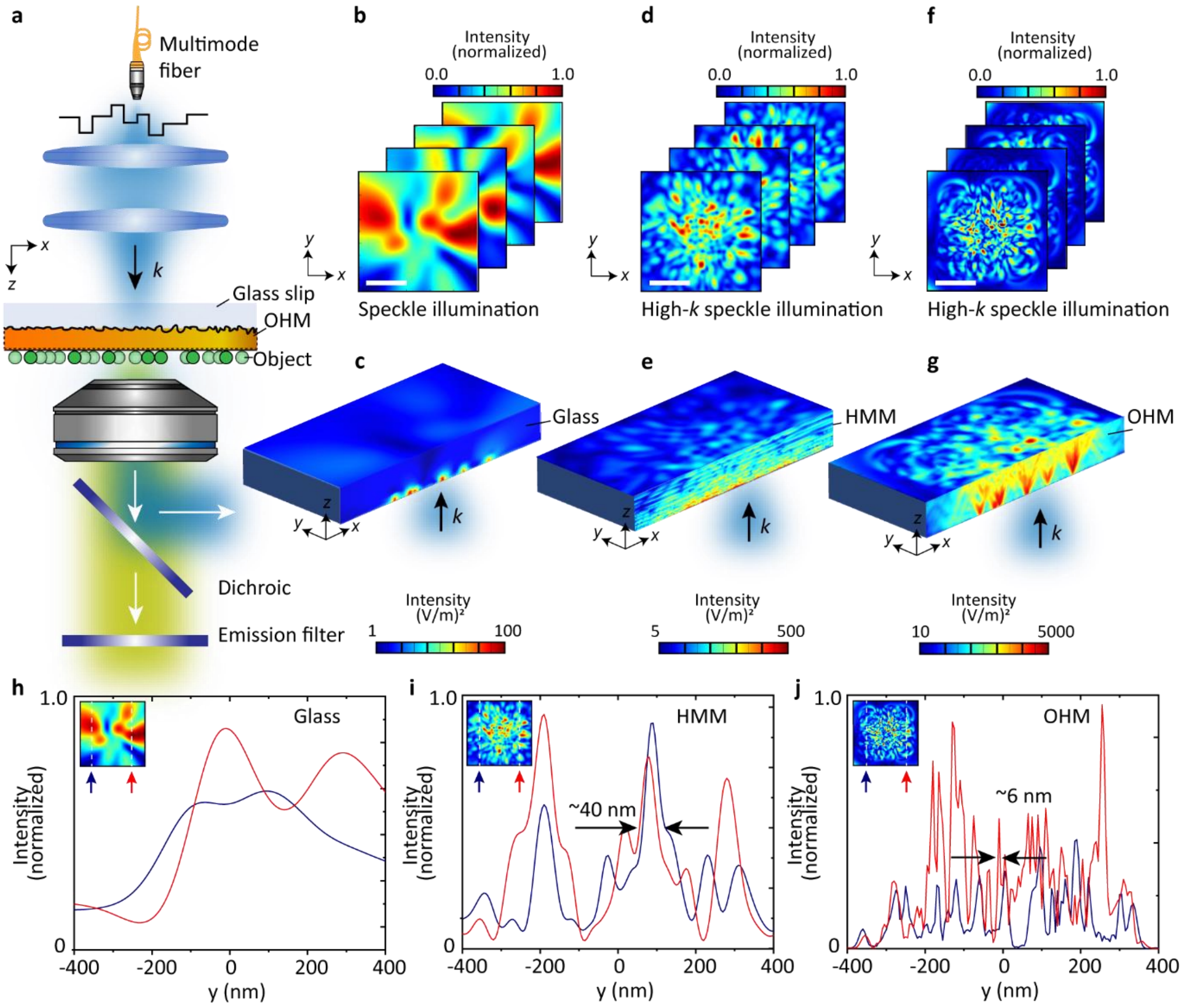

Fig. S5 | Speckle illumination microscopy using OHM. (a) Multimode fiber coupled excitation laser beam (488 nm) propagates back from the sample and is focused on the sample plane. After passing through an emission filter, the fluorescence signal is collected by a sCMOS camera. (b, c) Conventional speckle illumination pattern. (d, e) High-*k* speckle illumination pattern generated by the multilayer HMM (Ag/SiO_2, 50% metal filling ratio, 9 pairs) for unit cell size P = 20 nm. (f, g) High-*k* speckle illumination pattern generated by the OHM at the object plane. Scale bar: 500 nm. (h-j) The cross-section curves.

To show the conceptual features of high-*k* speckles on the top surface of an OHM, the speckles represented by the field intensity distribution at the distance 10 nm above the top surface of the OHM are simulated with the finite difference time domain (FDTD) method. To generate random speckles resulting from the scattering by impurities, surface roughness, and grain boundaries at the bottom surface of OHM, randomly oriented point dipole sources with random initial phases are used at the bottom of the substrate. We compare the near-field illumination speckles on the sample plane generated by a glass (Figs. S5b,c), a multilayer HMM (Ag/SiO_2, 50% metal filling ratio, 9 pairs) with unit cell size P = 20 nm (Figs. S5d,e), and the OHM substrate (Figs. S5f,g). As can be seen, the speckle generated from the OHM shows much finer and strongly confined high-intensity patterns with a higher spatial-frequency (Figs. S5h,i,j). The cut-off spatial frequency of the speckle patterns was calculated using the fast Fourier transform in the spatial-frequency domain, which is ~1.5k_0, ~7k_0, and ~50k_0 for the glass (Figs. S4b,c), the HMM (Figs. S5d,e), and the OHM (Figs. S5f,g), respectively. The generated speckles can be controlled by tuning the incident non-uniform illumination patterns. In the experimental demonstration, the high-*k* speckle illuminations were varied by adding mechanical forces on the multi-mode fiber with a stepping motor. At the sample plane, the high-contrast and high-*k* speckles excited the fluorophores in a specimen, and after passing through an emission filter (520/40 nm band-pass filter), the fluorescence signal was collected by a sCMOS camera.

FDTD Simulation. Perfectly matched layer (PML) boundary conditions were set up in the *x*-, *y*- and *z*-direction. For the permittivity of the OHM, we employed the values obtained from the VASE measurement. A minimum mesh step size of 0.25 nm was defined. To generate random speckle illumination, randomly oriented point dipole sources λ = 488 nm with random initial phases are used at the bottom of the substrate.

S6. SEM images of Cos-7 cells

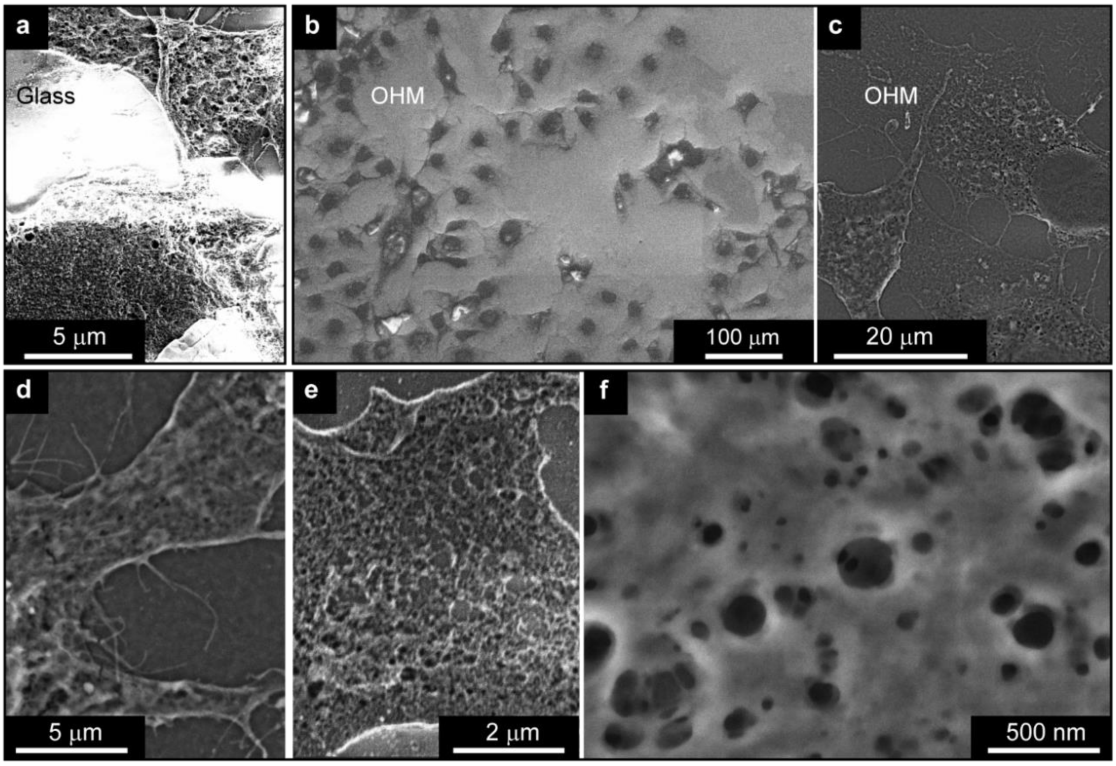

Fig. S6 | Scanning electron microscopy (SEM) images of Cos-7 cells cultured on glass coverslips and OHM substrates. SEM images of Cos-7 cells (a) on glass coverslips and (b-f) OHM substrate. The SEM images are taken under the same accelerating voltage. Under SEM, the glass sample leads rapidly accumulated charges, resulting in overly bright and unstable signals.

References

[1] S. V. Zhukovsky, O. Kidwai, J. E. Sipe, *Opt. Express* **2013**, *21*, 14982.
[2] W. Yan, M. Wubs, N. A. Mortensen, *Phys. Rev. B* **2012**, *86*, 205429.
[3] B. Wood, J. B. Pendry, D. P. Tsai, *Phys. Rev. B* **2006**, *74*, 115116.
